# Baseline MRI habitat imaging for predicting treatment response to neoadjuvant chemoradiotherapy in locally advanced rectal cancer

**DOI:** 10.3389/fonc.2025.1551224

**Published:** 2025-07-11

**Authors:** Muzhen He, Huijian Chen, Chao Xu, Zhibo Wu, Zijie Lin, Yang Song, Guang Yang, Mingping Ma, Fangqin Xue

**Affiliations:** ^1^ Department of Radiology, Shengli Clinical Medical College of Fujian Medical University, Fujian Provincial Hospital, Fuzhou University Affiliated Provincial Hospital, Fuzhou, China; ^2^ Department of Gastrointestinal Surgery, Shengli Clinical Medical College of Fujian Medical University, Fujian Provincial Hospital, Fuzhou University Affiliated Provincial Hospital, Fuzhou, China; ^3^ MR Scientific Marketing, Siemens Healthineers Ltd., Shanghai, China; ^4^ Shanghai Key Laboratory of Magnetic Resonance, East China Normal University, Shanghai, China

**Keywords:** habitat, locally advanced rectal cancer (LARC), neoadjuvant chemoradiotherapy (NCRT), radiomics, magnetic resonance imaging (MRI)

## Abstract

**Purpose:**

This study was to assess whether baseline magnetic resonance habitat imaging can predict the efficacy of neoadjuvant chemoradiotherapy (nCRT) in patients with locally advanced rectal cancer (LARC).

**Methods:**

This retrospective study analyzed data from 181 patients with locally advanced rectal cancer, including 60 who exhibited a good treatment response. The cohort was randomly divided into a training set (127 patients, 42 with good response) and a validation set (54 patients, 18 with good response). Five models were developed: Model_Clinic_, Model_Radiomics_, Model_Habitat_, Model_Clinic+Radiomics_, and Model_Clinic+Habitat_. Model performance was assessed using the area under the receiver operating characteristic (ROC) curve (AUC) for both training and validation sets.

**Results:**

The AUC values for predicting the efficacy of LARC neoadjuvant therapy were as follows: in the training set, Model_Clinic_ achieved 0.788, Model_Radiomics_ 0.827, Model_Habitat_ 0.815, Model_Clinic+Radiomics_ 0.938, and Model_Clinic+Habitat_ 0.896; in the test set, the corresponding AUCs were 0.656, 0.619, 0.636, 0.532, and 0.710, respectively. Decision curve analysis demonstrated that the clinical combined habitat model (Model_Clinic+Habitat_) provided higher net benefits than other models within a threshold probability range of 20% to 80%.

**Conclusion:**

The habitat model we developed, which integrates first-order and clinical features, demonstrates potential for predicting the efficacy of nCRT clinically interpretable spatial heterogeneity information. This model may aid in personalized treatment decision-making for LARC.

## Introduction

According to the GLOBOCAN 2022 data, colorectal cancer is the third most commonly diagnosed cancer and the second leading cause of cancer-related mortality, with over 1.9 million new cases accounting for 9.6% of all new cancer cases. The incidence of rectal cancer representing 30-50% of colorectal cancer cases (1), continues to rise. Approximately half of the cases are diagnosed as locally advanced rectal cancer (LARC) at initial presentation (2). LARC is defined as a rectal tumor located within 15 cm of the anal verge and classified as stage T3, T4, or N+ with M0 based on the American Joint Committee on Cancer (AJCC) staging system (3). In recent years, preoperative neoadjuvant chemoradiotherapy (nCRT), followed by total mesorectal excision (TME) has emerged as standard treatment of LARC, resulting in significant tumor regression and pathological downstaging (4).

In clinical practice, the pathological response of patients with LARC undergoing nCRT exhibits significant heterogeneity, with only approximately 13%-22.2% achieving a pathological complete response (pCR), defined as no residual tumor cells in the resected specimen (5, 6). For patients with a clinical complete response (cCR) before radical surgery and no evidence of local tumor residue after nCRT confirmed by physical and ancillary examinations, a “watch-and-wait” strategy may be considered under strict follow-up to avoid the risks of radical surgery (7, 8). Conversely, patients with a poor response to nCRT not only fail to achieve favorable therapeutic outcomes but may also experience treatment-related toxicities (9). Currently, postoperative pathology remains the gold standard for assessing nCRT efficacy in rectal cancer, while magnetic resonance imaging (MRI) is widely used for staging, treatment evaluation, and follow-up (10).

Radiomics involves extracting and analyzing a large number of advanced quantitative imaging features from routine radiological examinations (11). Previous studies have demonstrated its significant contributions to precise staging, treatment efficacy evaluation, genetic status assessment, and prognosis prediction in colorectal cancer (12, 13). However, tumors are complex ecosystems composed of heterogeneous subpopulations of tumor cells with varying invasive potential, growth rates, drug sensitivity, and prognosis outcomes (14). Conventional radiomics, which predominantly evaluates the entire tumor region, has limitations as it fails to account for spatial heterogeneity within the tumor.

Habitat imaging analysis is an innovative radiomics approach that identifies tumor subregions with similar characteristics using quantitative imaging markers (15). Preliminary findings suggest that habitat analysis holds promise for predicting molecular profiles, treatment response, and prognosis in diseases such as glioblastoma and breast cancer, while also quantifying intra-tumor heterogeneity (ITH) (16–18). For example, Chao et al. (19) demonstrated that regions with high cellular density and low arrangement complexity in breast cancer, identified through intravoxel incoherent motion (IVIM) and diffusional kurtosis imaging (DKI), were more likely to achieve a pCR. Although habitat imaging has shown potential in other malignancies, its application in predicting treatment efficacy for rectal cancer remains unexplored. This study aims to evaluate the predictive accuracy and clinical utility of baseline MR habitat imaging models for assessing nCRT response in locally advanced rectal cancer.

## Methods

### Study setting and timeframe

A retrospective analysis was performed on 181 patients with rectal cancer who received treatment at Fujian Provincial Hospital from June 2020 to June 2024. The study protocol was approved by the Fujian Provincial Hospital Ethics Committee (approval code: K2021-05-007; May 2019), and all procedures were conducted in compliance with relevant guidelines and regulations. Written informed consent was obtained from all participating patients.

**Figure 1:**
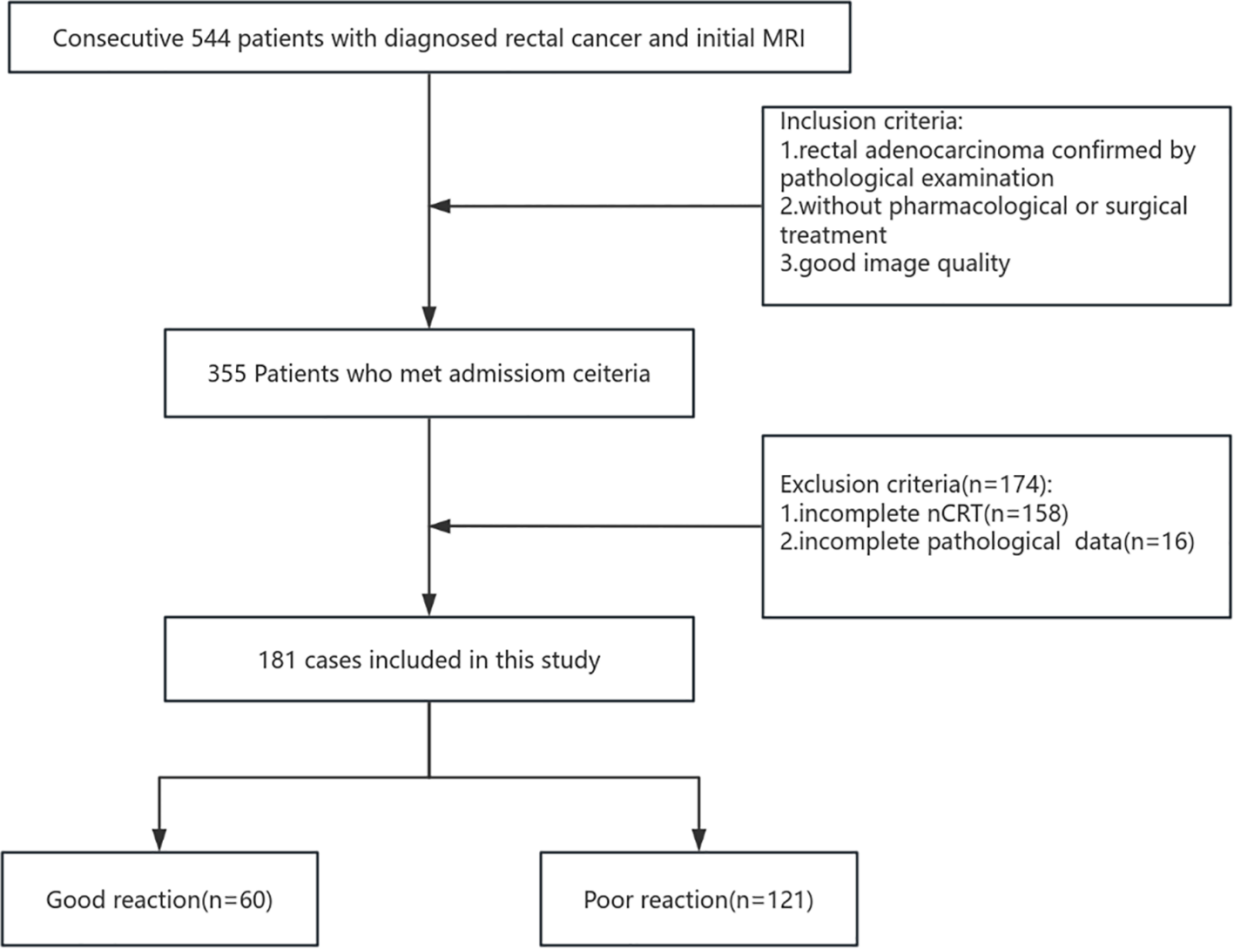
Flowchart depicting patient selection and grouping.

The inclusion criteria were as follows: (I) clinically diagnosed LARC with the tumor located ≤15 cm from the anal verge; (II) no prior pharmacological or surgical treatment; (III) completion of neoadjuvant chemoradiotherapy at our institution; (IV) availability of complete pathological data; and (V) availability of high-quality MRI with complete criteria included: (I) incomplete neoadjuvant therapy at our institution, (II) baseline distant metastasis, (III) significant MRI artifacts or incomplete sequences compromising habitat analysis, or (IV) previous pelvic radiotherapy or rectal resection. **Figure 1** illustrates the patient selection and grouping process.

The dataset was randomly divided into training (n=127) and test (n=54) cohorts in a 7:3 ratio. The training cohort was used to develop the diagnostic model, while the test cohort served to evaluate model performance.

### Neoadjuvant treatment

The intensity-modulated radiation therapy regimen consisted of two schedules: short-course radiotherapy delivering a total dose of 25 Gy in 5 Gy fractions, and long-course chemoradiation involving 22–25 fractions of 2–2.3 Gy to the gross tumor volume and 1.8–2.0 Gy to the clinical target volume. Neoadjuvant treatment modalities included long-course radiotherapy with concurrent chemotherapy (n=19), short-course radiotherapy followed by neoadjuvant chemotherapy (n=11), long-course radiotherapy followed by neoadjuvant chemotherapy (n=61), and neoadjuvant chemotherapy alone (n=90).

### Clinical characteristics

The clinical baseline characteristics included the following parameters: age, sex, type of therapy (whether combined with radiation therapy), tumor location, maximum tumor thickness, maximum tumor length, distance to the anal verge, presence of extramural vascular invasion (EMVI), mesorectal fascia (MRF) status, histologic grade, MRI-predicted T/N stage (MRI-T/N stage) (3), and pretreatment levels of carcinoembryonic antigen (CEA), alpha-fetoprotein (AFP), carbohydrate antigen 125 (CA125), carbohydrate antigen 724 (CA724), and carbohydrate antigen 199 (CA199).

### Pathologic assessment of response

Pathological assessment and postoperative TNM restaging were performed according to the pathological findings of the surgically resected specimens. Tumor regression response was evaluated using the AJCC Tumor Regression Grade (TRG) system, which categorizes responses as follows: Grade 0 (complete response) indicates no viable tumor cells; Grade 1 (moderate response) denotes residual small clusters or single tumor cells; Grade 2 (minimal response) represents residual tumor with predominant fibrosis; and Grade 3 (poor response) shows minimal or no tumor regression with extensive residual cancer. The specimens were reviewed by an experienced pathologist (Y.H.) with 20 years of expertise in rectal cancer diagnosis. TRG 0–1 was considered a good response, whereas TRG 2–3 was classified as a poor response.

### Imaging studies

Prior to MRI examination, patients were instructed to fast for 4–6 hours and empty their bladder and bowels. The scans were performed using three 3T MRI scanners (MAGNETOM Prisma, Skyra, and Verio; Siemens Healthcare, Erlangen, Germany) with a 16-channel phased-array surface coil. The imaging protocol included high-resolution axial T2-weighted imaging (T2WI) and diffusion-weighted imaging (DWI), with apparent diffusion coefficient (ADC) maps automatically generated by the system. Detailed acquisition parameters are provided in **Table 1**.

**Table 1:** The imaging protocol of MR sequences.

Machine	Sequences	Protocol					
Machine1	T2WI	TR	4200ms	TE	92ms	Thickness	3.5mm
		Slices	20	Bandwidth	200Hz/Px	FOV	200mm
		FOV phase	100%	Matrix	384×288	Averages	2
		concatenations	1				
	DWI	TR	8100ms	TE	61ms	Thickness	3.5mm
		Slices	20	Bandwidth	2000Hz/Px	FOV	240mm
		FOV phase	88%	Matrix	100×100	scan time	186s
		B-values	50,600,1200s/mm^2^		Averages	1,2,3	
Machine2	T2WI	TR	4280ms	TE	92ms	Thickness	3.5mm
		Slices	20	Bandwidth	200Hz/Px	FOV	200mm
		FOV phase	100%	Matrix	384×288	Averages	2
		concatenations	1				
	DWI	TR	7000ms	TE	50ms	Thickness	3.5mm
		Slices	20	Bandwidth	2272Hz/Px	FOV	200mm
		FOV phase	100%	Matrix	100×100	scan time	203s
		B-values	50,600,1200s/mm^2^		Averages	1,3,4	
Machine3	T2WI	TR	4100ms	TE	96ms	Thickness	3.0mm
		Slices	30	Bandwidth	200Hz/Px	FOV	200mm
		FOV phase	100%	Matrix	320×224	Averages	2
		concatenations	2				
	DWI	TR	8400ms	TE	93ms	Thickness	3.0mm
		Slices	30	Bandwidth	1158Hz/Px	FOV	260mm
		FOV phase	85%	Matrix	160×120	scan time	260s
		B-values	0,800,1200s/mm^2^		Averages	4,4,4	

### Tumor segmentation

T2WI and ADC maps were visualized using 3D Slicer (version 4.10.2, www.slicer.org) for image segmentation. A radiologist (H.C.) with three years of experience in rectal cancer diagnosis performed the initial region of interest (ROI) delineation. All segmentations were manually drawn along the inner tumor capsule boundary on axial T2WI by experienced radiologists, excluding peritumoral edema, adjacent vessels, lymph nodes, and normal rectal walls to minimize contamination from surrounding tissues. For interobserver reproducibility assessment, 50 randomly selected cases from the training cohort were independently segmented by the same radiologist (H.C.) and another radiologist (M.H.) with 14 years of rectal cancer imaging experience. Reproducibility was evaluated using a two-way random absolute agreement intraclass correlation coefficient (ICC), where values >0.75 indicated good consistency, 0.50–0.75 moderate consistency, and <0.50 poor consistency.

### Feature extraction and habitat analysis

We used PyRadiomics (v3.0) to extract quantitative features from the ROI encompassing the entire tumor. First, we performed Z-score normalization on T2W images and applied a re-segmentation strategy with a 3-sigma restriction for both ADC and T2WI. For texture analysis, we discretized the images into 16 bins based on the comprehensive training dataset, using a 2.5-D merge strategy. We then extracted multiple feature categories, including first-order, shape, gray-level co-occurrence matrix, gray-level run length matrix, gray-level size zone matrix, gray-level dependence matrix, and neighboring gray tone difference matrix features. Additionally, we applied a wavelet transform with a coif1 filter to derive higher-dimensional features. In total, 1684 radiomics features were extracted.

In addition, we used K-means, an unsupervised clustering method, to segment the entire tumor ROI based on combined T2WI and ADC map data. Voxel values from these parametric maps were used to cluster the tumor into subsegments. The optimal number of clusters (K) for K-means was determined using the Calinski-Harabasz (CH) Score (20) (Appendix 1). For each segment, we extracted volume, volume ratio, and first-order features from the corresponding regions of each parametric map. During habitat feature extraction, a 3-sigma restriction was applied within the tumor ROI to exclude voxels with signal characteristics inconsistent with viable tumor tissue (e.g., luminal air or fecal residues). These features were used to assess tumor heterogeneity.

### Model building

**Figure 2:**
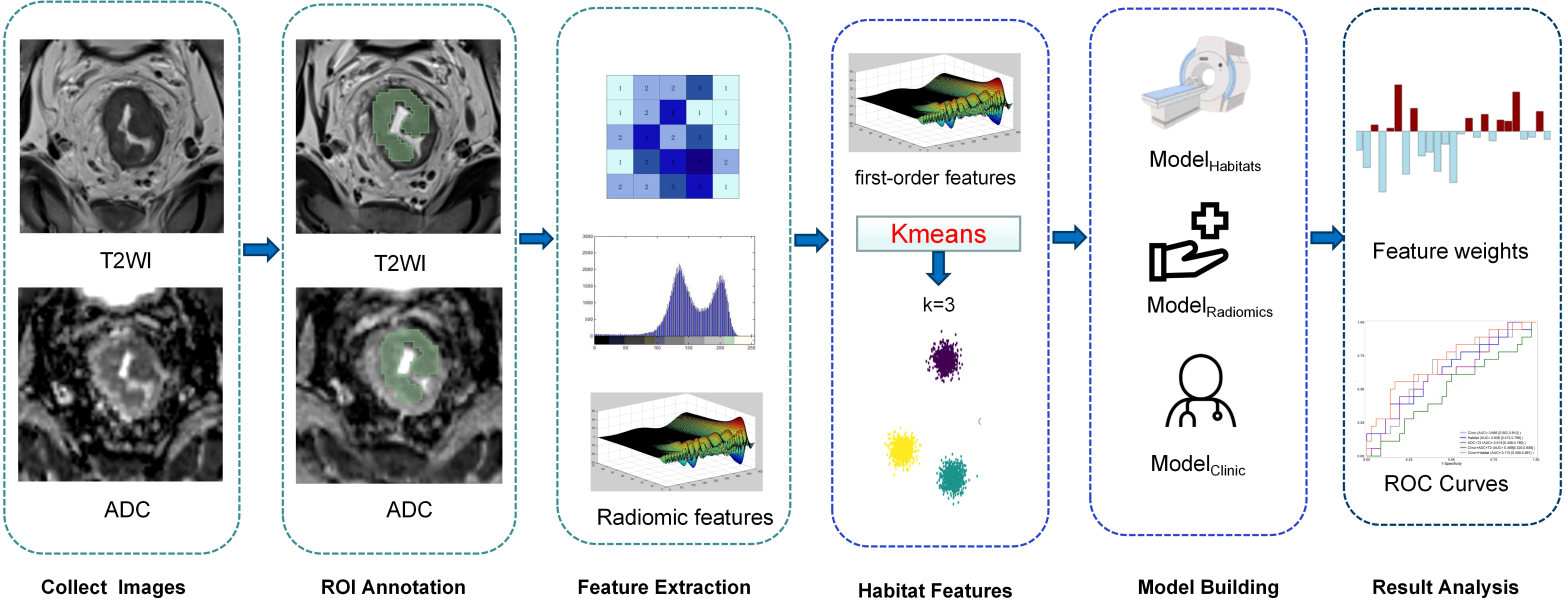
Workflow for establishment of the models.

Features with ICCs below 0.75 were excluded, and the remaining features were deemed stable. The selected features were normalized using the Z-score method in the training set. Following normalization, the Pearson correlation coefficient was applied with a threshold of 0.9 to evaluate collinearity between feature pairs, thereby reducing feature space dimensionality. The least absolute shrinkage and selection operator (LASSO) method was then used to identify the most predictive features from the training set. A radiomics score (Radscore) was calculated for each patient as a linear combination of the selected features, weighted by their respective coefficients. Two models were developed: one utilizing habitat features (Model_Habitat_) and the other employing radiomics features (Model_Radiomics_). The Radscore, representing the combined radiomic results for each patient, was computed as follows: Radscore=∑Ni=1ωi·χi+β, where N denotes the number of features, ω_i_ represents the feature weight, χ_i_ is the value of the i^th^ feature, and β is the bias term (5) (**Figure 2**).

### Statistical analysis

Statistical analyses were performed using R software (version 4.4.1). A stepwise logistic regression based on the Akaike information criterion was used to identify significant clinical variables and develop the clinical model. The LASSO method from the “glmnet” package was applied to construct the habitat and radiomics models, yielding the habitat score and radiomics score. All individual models (Model_Radiomics_, Model_Habitat_) employed LASSO regression with 10-fold cross-validation for feature selection and regularization. Logistic regression was used to integrate Model_Radiomics_, the clinical combined habitat model (Model_Clinic+Habitat_), and the clinical combined radiomics model (Model_Clinic+Radiomics_). Model performance was evaluated using the area under the receiver operating characteristic (ROC) curves and the mean area under the curve (AUC). Sensitivity, specificity, positive predictive value (PPV), and negative predictive value (NPV) were calculated based on the maximum Yorden Index of the training cohort. The DeLong test was used to compare model performance, and decision curve analysis (DCA) was performed to assess net benefit rates across prediction thresholds. A two-tailed P-value <0.05 was considered statistically significant.

## Results

### Characteristics of the study sample

A total of 181 patients diagnosed with LARC were included in this retrospective study based on the selection criteria, among whom 60 exhibited a good treatment response. The patients were randomly divided into a training set (n = 127, including 42 good responders) and a test set (n = 54, including 18 good responders). No significant differences in clinical characteristics were observed between the training and test sets (P > 0.05), as shown in **Table 2**.

**Table 2:** Characteristics of patients in the training and test sets (n = 181).

Characteristic	Training set (n=127)	Test sett (n=54)	*P* value
Age(yrs),mean ± SD	60.59 ± 9.71	58.98 ± 10.29	0.318
Gender, n(%)			0.191
Man	88 (69.29)	32 (59.26)	
Female	39 (30.71)	22 (40.74)	
Location,n(%)			0.691
Low	41 (32.28)	14 (25.93)	
Median	68 (53.54)	32 (59.26)	
High	18 (14.17)	8 (14.81)	
T,n(%)			0.348
T3	80 (62.99)	30 (55.56)	
T4	47 (37.01)	24 (44.44)	
N,n(%)			0.676
N0	16 (12.60)	5 (9.26)	
N1	31 (24.41)	16 (29.63)	
N2	80 (62.99)	33 (61.11)	
Thickness(mm),mean ± SD	18.95 ± 7.35	18.63 ± 6.87	0.783
Length(mm),mean ± SD	50.98 ± 17.09	53.57 ± 21.92	0.394
EMVI,n(%)			0.89
Positive	79 (62.20)	33 (61.11)	
Negative	48 (37.80)	21 (38.89)	
MRF,n(%)			0.503
Positive	78 (61.42)	36 (66.67)	
Negative	49 (38.58)	18 (33.33)	
CEA,n(%)			0.525
Positive	64 (50.39)	30 (55.56)	
Negative	63 (49.61)	24 (44.44)	
AFP	2.79 ± 1.64	2.74 ± 1.27	0.836
CA199	64.91 ± 238.62	69.61 ± 184.79	0.897
CA724	4.71 ± 11.71	8.85 ± 19.66	0.081
CA125	10.92 ± 5.70	12.56 ± 6.33	0.089
Differentiation,n(%)			0.519
Poor	3 (2.36)	3 (5.56)	
Medium-high	124 (97.64)	51 (94.44)	
Therapy			0.782
with radiation therapy	63 (49.61)	28 (51.85)	
without radiation therapy	64 (50.39)	26 (48.15)	

Only variables with a *P* value less than 0.05 were considered significant. EMVI (extramural vascular invasion), MRF, mesorectal fascia; CEA, carcinoembryonic antigen; AFP, alpha-fetoprotein; CA125, carbohydrate antigen 125; CA724, carbohydrate antigen 724; CA199, carbohydrate antigen 199.

### Feature screening

The extracted features were analyzed using the LASSO algorithm for dimensionality reduction of eigenvalues. Features with non-zero coefficients were selected for subsequent analyses (Appendix 2). The clinical model incorporated maximum tumor length, MRI-N stage, CEA levels, therapy, and MRF.

### Performance of the models and model comparison

**Figure 3:**
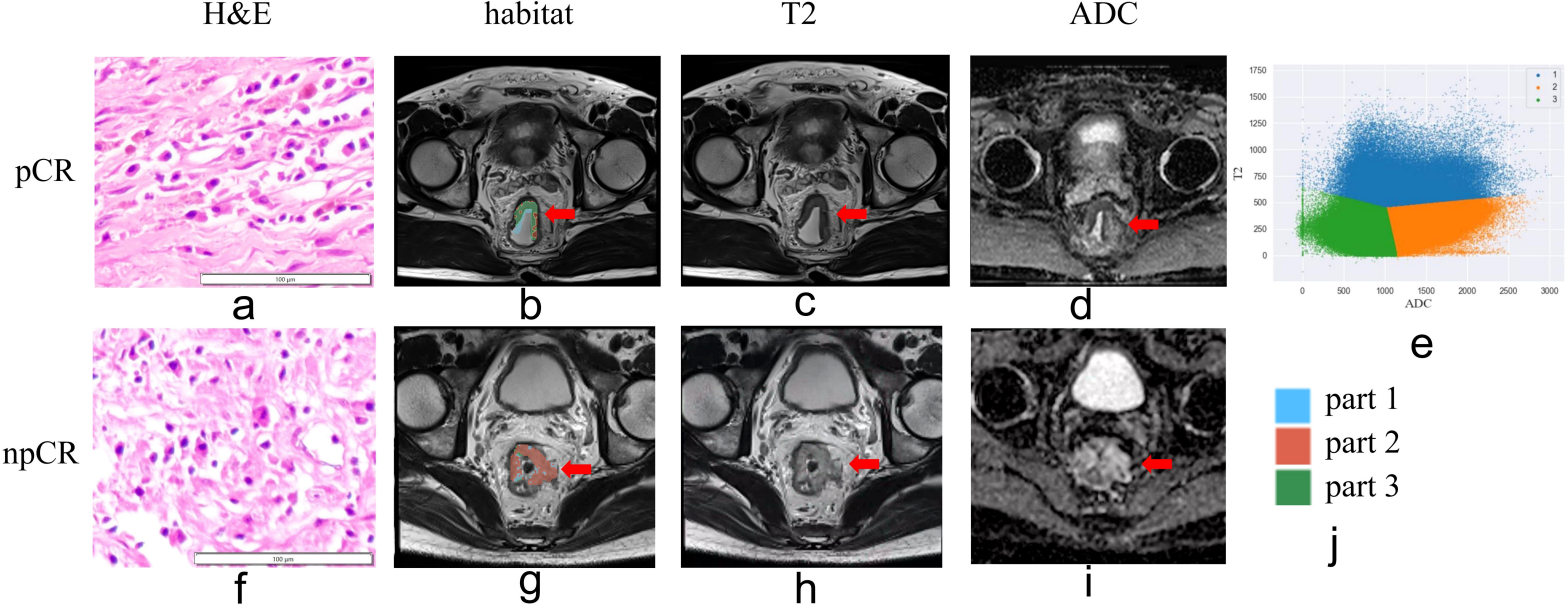
Habitat imaging of patients with and without pathologic complete response (pCR) following neoadjuvant chemoradiotherapy. **(a, e)** Hematoxylin-eosin staining (×10). **(b, g)** Habitat imaging. **(c, h)** T2WI. **(d, i)** ADC map. **(e, j)** Voxel distribution in regions of interest (ROIs) on T2WI and ADC maps (K = 3). Part 1 stands for region of high-T2-signal; part 2 stands for region of low-T2-signal and high-ADC-value; part 3 stands for region of low-T2-signal and low-ADC-value region areas. In tumors without pCR, the predominant regions exhibited low-T2-signal and high-ADC-value characteristics.

**Figure 4:**
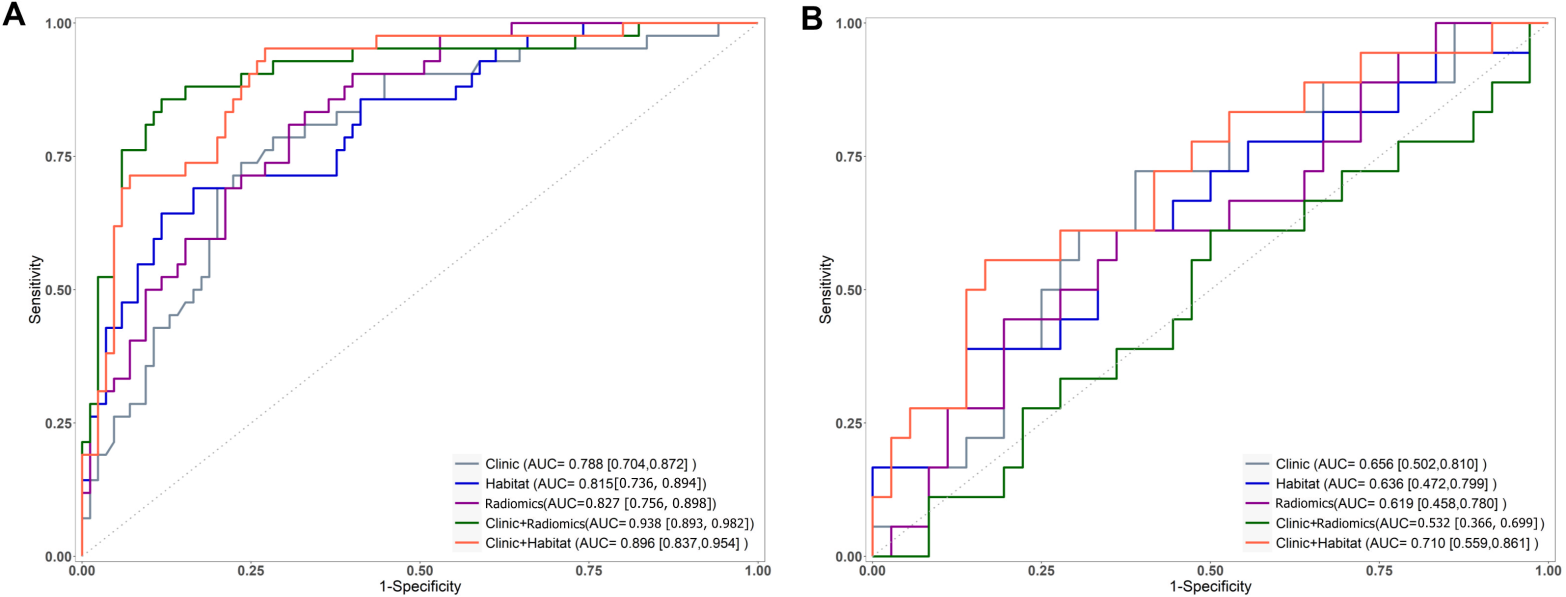
Receiver operating characteristic curves for models predicting the efficacy of nCRT in LARC on the training **(A)** and test **(B)** sets, where ADC+T2WI represents the Model_Radiomics_).

The AUC for Model_Clinic_ was 0.788 in the training set and 0.656 in the testing set. In the testing set, Model_Clinic_ demonstrated a predictive accuracy of 0.648, sensitivity of 0.722, specificity of 0.611, PPV of 0.481, and NPV of 0.815. For Model_Radiomics_, the AUC values were 0.827 in the training set and 0.619 in the testing set. In the testing set, Model_Radiomics_ exhibited a predictive accuracy of 0.630, sensitivity of 0.611, specificity of 0.639, PPV of 0.458, and NPV of 0.767. Model_Habitat_, utilizing T2 and ADC maps with clustering at K = 3, achieved the highest AUC in the test set. The whole-tumor ROI was classified into three regions: high-T2-signal, low-T2-signal and high-ADC-value, and low-T2-signal and low-ADC-value (**Figure 3**). Tumors with poor response to LARC primarily displayed low-T2-signal and high-ADC-value regions, whereas those with good response exhibited high-T2-signal and low-ADC-value regions. Model_Habitat_ yielded an AUC of 0.815 in the training set and 0.636 in the test set, with predictive accuracy, sensitivity, specificity, PPV, and NPV values of 0.704, 0.389, 0.861, 0.583, and 0.738, respectively, as summarized in **Table 3** and **Figure 4**.

**Table 3:** Results of ROC curve analysis for predicting the efficacy of nCRT in LARC utilizing the following models: clinic, habitat, radiomics, clinic+radiomics, clinic+habitat.

Model	AUC (95%CI)	Accuracy	Sensitivity	Specificity	PPV	NPV	F1-Score
Clinic							
Train Set	0.788 (0.704, 0.872)	0.740	0.786	0.718	0.579	0.871	0.667
Test Set	0.656 (0.502, 0.810)	0.648	0.722	0.611	0.481	0.815	0.575
Habitat							
Train Set	0.815 (0.736, 0.894)	0.787	0.690	0.835	0.674	0.845	0.681
Test Set	0.636 (0.472, 0.799)	0.704	0.389	0.861	0.583	0.738	0.466
Radiomics							
Train Set	0.827 (0.756, 0.898)	0.701	0.905	0.600	0.528	0.927	0.666
Test Set	0.619 (0.458, 0.780)	0.630	0.611	0.639	0.458	0.767	0.521
Clinic+Radiomics							
Train Set	0.938 (0.893, 0.982)	0.874	0.952	0.835	0.741	0.973	0.833
Test Set	0.532 (0.366, 0.699)	0.611	0.389	0.722	0.412	0.703	0.398
Clinic+Habitat							
Train Set	0.896 (0.837, 0.954)	0.803	0.952	0.729	0.635	0.969	0.761
Test Set	0.710 (0.559, 0.861)	0.741	0.556	0.833	0.625	0.789	0.617

AUC, area under the curve; CI, confidence interval; nCRT, neoadjuvant chemoradiotherapy; ROC, receiver operating characteristic; LARC, locally advanced rectal cancer; PPV, Positive predictive value; NPV, Negative predictive value.

The AUC values for Model_Clinic+Radiomics_ were 0.938 in the training set and 0.532 in the testing set. In the test set, ModelClinic+Radiomics demonstrated a predictive accuracy of 0.611, sensitivity of 0.389, specificity of 0.722, PPV of 0.412, and NPV of 0.703. For Model_Clinic+Habitat_, the AUCs were 0.896 in the training set and 0.710 in the testing set. In the test set, ModelClinic+Habitat achieved a predictive accuracy of 0.741, with a sensitivity of 0.556, specificity of 0.833, PPV of 0.625, and NPV of 0.789, as detailed in **Table 3** and **Figure 4**.

**Figure 5:**
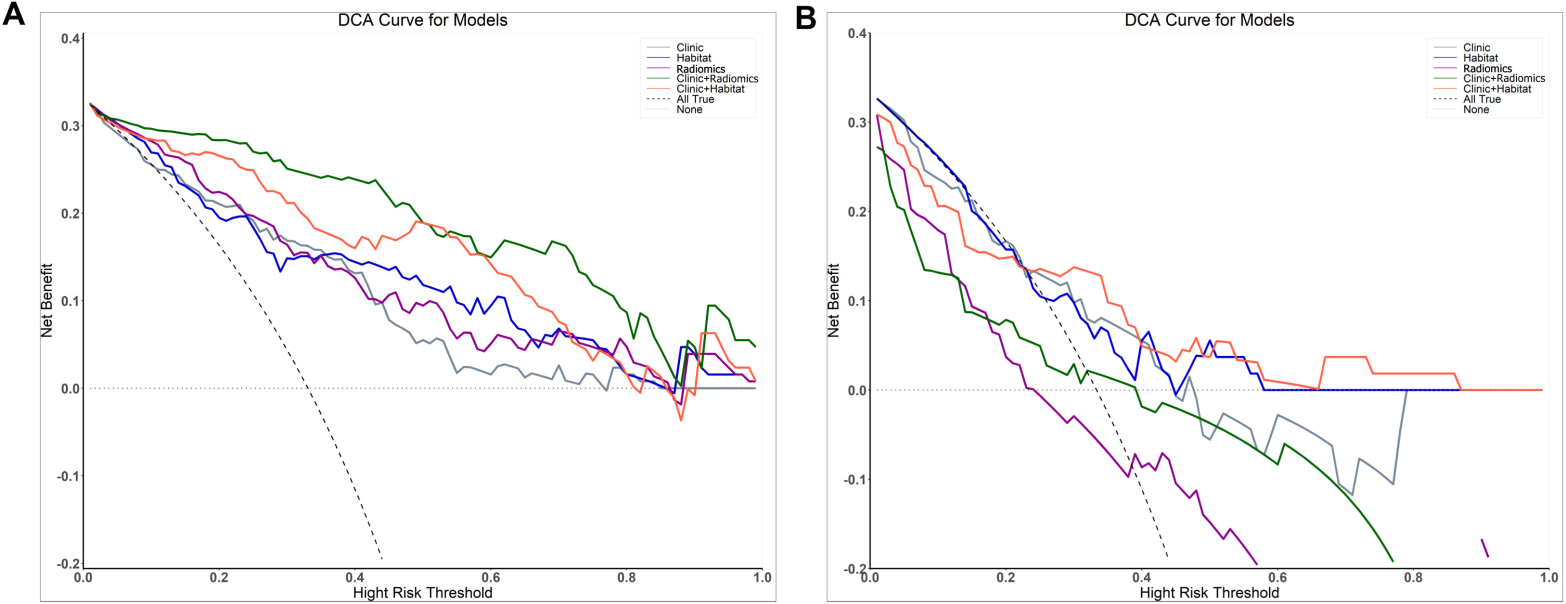
Decision curve analysis evaluating the performance of the prediction models on both the training **(A)** and test **(B)** sets.

The DeLong test showed no significant difference in performance between Model_Clinic_ and the other models (P > 0.05) in the test set. Furthermore, decision curve analysis (DCA) indicated that Model_Clinic+Habitat_ provided a net benefit increase of 0.05–0.12 over the clinically relevant threshold probability range of 0.2–0.8 compared to alternative models (**Figure 5**).

## Discussion

The study aimed to develop a model that integrates habitat analysis and clinical features to accurately predict the efficacy of nCRT for LARC. Habitat analysis served as a key component by enabling quantitative assessment of the spatial-temporal heterogeneity across the entire tumor, effectively providing a “virtual biopsy”.

In recent years, radiomics techniques for high-throughput extraction of quantitative image features from medical images have become a prominent research focus (21). In this study, the radiomics model achieved an AUC of 0.619, accuracy of 0.630, sensitivity of 0.611, specificity of 0.639, PPV of 0.458, and NPV of 0.767 on the test set. Tumors represent complex ecosystems comprising diverse subpopulations of tumor cells that continuously adapt to selective pressures within the tumor microenvironment (TME), including hypoxia, acidity, and regional cytokine heterogeneity. The TME facilitates tumor growth, contributing to ITH. ITH plays a critical role in enabling tumors to evade immune surveillance and develop therapeutic resistance, which is closely associated with tumor progression and prognosis (22).

Habitat imaging, based on Darwinian evolutionary dynamics, clusters tumor cell populations with similar characteristics as indicated by quantitative imaging markers to better depict ITH (23). The resulting subregions reflect diverse environmental selective pressures and cellular adaptive variations within the tumor. This approach establishes a clear and predictable association between macroscopic tumor features observed on imaging and the molecular, cellular, and microenvironmental characteristics of the microscopic cancer cell population (24–26). Our findings demonstrate that habitat imaging is useful for predicting the efficacy of neoadjuvant therapy in LARC.

In this study, habitat analysis incorporated first-order imaging histological features derived from T2WI and ADC maps. Our results demonstrate that tumor regions exhibiting low ADC and high T2WI values are more likely to respond favorably to treatment, whereas areas with high ADC and low T2WI values tend to show poorer response to nCRT. High ADC values typically reflect necrotic tissue with low cellularity, while low ADC values correspond to viable tumor regions with high cellular density (27). This correlation arises because densely proliferating tumor cells reduce extracellular space, restricting water molecule diffusion and consequently lowering ADC values. Conversely, necrotic tumor tissue often exists in hypoxic environments with slow metabolic activity, rendering it less susceptible to chemotherapeutic agents (27). Additionally, elevated T2 signal intensity may indicate increased tumor vascularity or edema, potentially improving drug delivery and contributing to better treatment outcomes.

Habitat models constructed solely with first-order histogram features offer a more accurate representation of biological characteristics than whole-tumor models. Incorporating texture features requires additional image processing steps, such as image discretization and texture-specific parameters (e.g., texture calculation direction), making texture features sensitive to a wider range of variables. In contrast, first-order histogram features depend only on voxel intensity (28), enhancing their intrinsic stability, robustness, and reproducibility while reducing susceptibility to noise and variations in image acquisition parameters (29). A hybrid model combining clinical and habitat features was also developed to improve interpretability and radiologist acceptance For comparative purposes, secondary feature selection was intentionally omitted in the combined models (Model_Clinic+Radiomics_ and Model_Clinic+Habitat_) to maintain a standardized framework. Although this approach allowed some overfitting in Model_Clinic+Radiomics_ it objectively demonstrated that Model_Clinic+Habitat_(train AUC 0.896, test AUC 0.710) inherently generalizes better than Model_Clinic+Radiomics_ (train AUC 0.938, test AUC 0.532) under identical modeling conditions. The pronounced overfitting in Model_Clinic+Radiomics_ highlights the superior robustness and translational reliability of the habitat-based approach, reinforcing its value as a stable predictor in our experimental design.

The DeLong test showed no statistically significant differences between models in the test set (P > 0.05). Madel_Habitat_ provides unique value by offering interpretable spatial heterogeneity insights, such as identifying subregions associated with treatment resistance. DCA demonstrated that Model_Clinic+Habitat_ yielded higher clinical net benefit across the threshold probability range of 0.2–0.8 compared to alternative models, supporting its practical utility despite the absence of statistical significance. In the test cohort, Model_Clinic+Habitat_ achieved a specificity of 0.833 and an AUC of 0.710. The high specificity indicates robust performance in correctly identifying patients with favorable treatment responses (minimizing false negatives), which is clinically important for preventing undertreatment. For example, when the model predicts a positive response, clinicians can be 83.3% confident that treatment intensification may not be necessary. Although the AUC of 0.710 reflects moderate overall discriminative ability, its integration with DCA confirms meaningful clinical utility: within the 0.2–0.8 threshold range, the model may reduce 5–12 unnecessary treatments per 100 patients using the habitat-guided strategy—particularly valuable for avoiding overtreatment in likely responders or undertreatment in non-responders.

## Limitations

This study has several limitations. First, it is a single-center retrospective study with a relatively small sample size, and the developed model has not undergone external validation. However, our models trained with data from various MRI machines may exhibit greater robustness and generalizability due to exposure to a broader spectrum of variability in imaging protocols, hardware characteristics, and scanner-related artifacts. Additionally, our habitat analysis focused on stable first-order histogram features from fundamental sequences (T2WI and ADC), which are less susceptible to variability than higher-order texture features. Nevertheless, external validation in a multi-center cohort is essential to confirm the model’s real-world applicability. Future work will prioritize such validation and explore harmonization techniques to improve generalizability. Second, the model incorporated only T2WI and ADC sequences, prioritizing clinical translatability and stability. While multi-parametric habitat models integrating IVIM or DCE-MRI could enhance tumor microenvironment characterization, their omission was due to practical considerations in this retrospective study. Future research should integrate these advanced sequences to develop more comprehensive models. Lastly, despite sample size constraints and class imbalance, our approach included rigorous safeguards: feature stability (ICC >0.75), LASSO regularization for model complexity control, and AUC-based performance evaluation. Future validation in larger, prospectively balanced cohorts will incorporate advanced imbalance-handling strategies to improve clinical utility.

## Conclusion

Our findings indicate that baseline MRI habitat imaging reveals tumor heterogeneity, suggesting differential treatment responses across various tumor regions. Notably, areas with specific imaging markers exhibit a higher likelihood of favorable response to nCRT. Compared to conventional radiomics models, our integrated habitat imaging and clinical feature model demonstrates improved interpretability and enhanced clinical utility, as evidenced by DCA within critical decision thresholds. Although the habitat model achieved clinically useful performance (AUC 0.710) rather than diagnostic superiority, its capacity to delineate spatially distinct tumor subregions provides actionable insights beyond traditional radiomics. This approach holds promise as a valuable tool for personalized treatment decision-making in locally advanced rectal cancer.

## Data Availability

The raw data supporting the conclusions of this article will be made available by the authors, without undue reservation.
