# Supplementary material for: Baseline MRI habitat imaging for predicting treatment response to neoadjuvant chemoradiotherapy in locally advanced rectal cancer
Source: Front Oncol. 2025 Jul 11;15:1551224. doi: 10.3389/fonc.2025.1551224 (PMC12289506; doi:10.3389/fonc.2025.1551224)
Supplement: Supplementary file 1 [file DataSheet1.pdf]

Appendix 1

(1) We tried to determine the number of habitats using a bootstrap approch. The specific steps are as follows:

The data were randomly sampled repeatedly with replacement till sample size was the same as that of the original dataset. Then the sampled dataset was used to calculate the CH index. This process was repeated 50 times, and then the average and standard deviation of CH index were calculated. Then we applied the one standard error rule (1-SE) to select the minimum k that produced an average CH index greater then the maximum average CH index minus one standard error of the corresponding CH index (Figure S-1). Furthermore, we also found the standard deviation of the CH index is minimum when k=3.

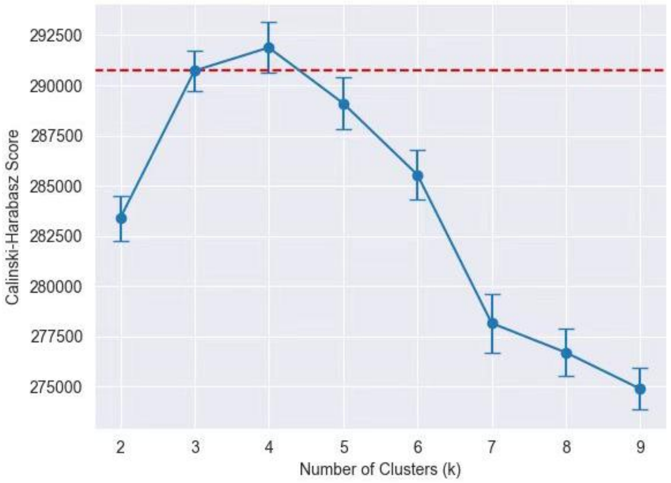

Figure S-1: The plot of the Calinski-Harabasz score against to the cluster K number

(2) As shown in the scatter plots, when K = 4 was used, the meaning of part 4 was more difficult to explain than that of the other parts. On the other hand, when K = 3 was used, each part had a clear meaning (Figure S-2).

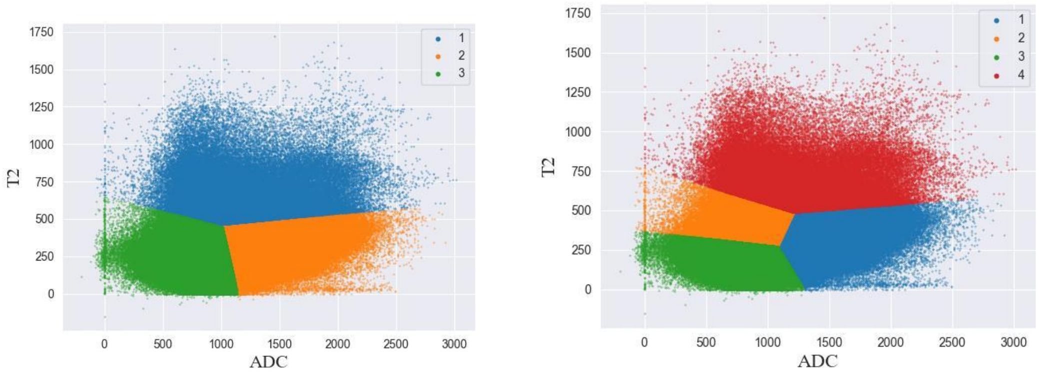

Figure S-2: The scatter plots (left: K = 3; right: K = 4)

Appendix 2

Quantitative features were extracted from ROIs containing whole tumours using PyRadiomics (v3.0). In total, 120 habitat variables, 1684 ADC variables and 1684 T2 variables were extracted. The extracted variables were normalised and included in the LASSO regression for variable selection. Twenty-five habitat variables, seven ADC variables and three T2WI variables were obtained after screening. The screened imaging features and their corresponding regression coefficients were weighted linearly combined to radiomics lables and habitat labels.

Risk factors and their coefficient values of habitat(A,B),ADC(C,D),and T2WI(E,F) selected using LASSO Cox regression analysis.

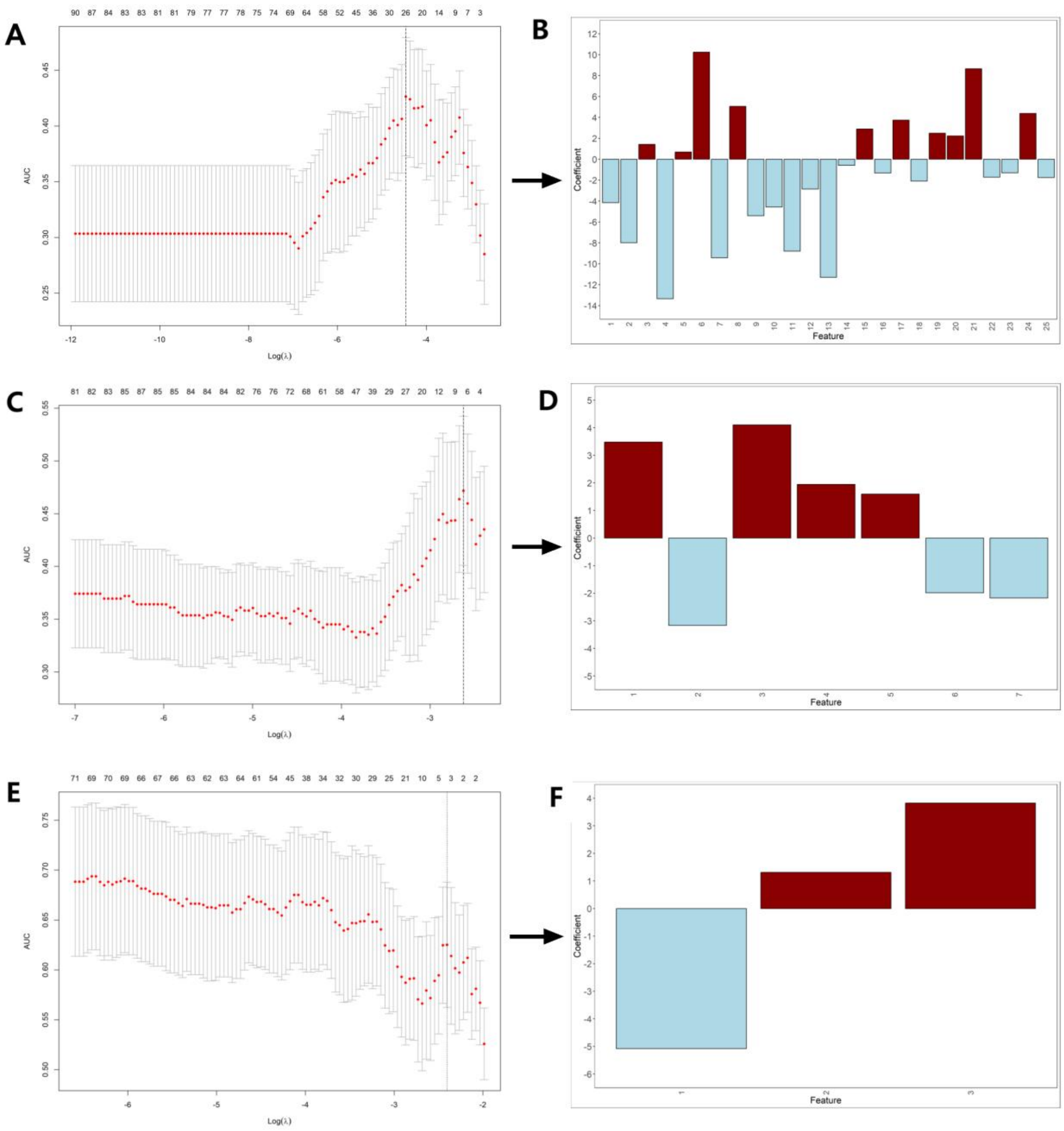

Features in figure a are shown from left to right:

ADC_mask1_Kurtosis、ADC_mask1_RobustMeanAbsoluteDevuation、ADC_mask1_Skewness、ADC_mask1_Uniformity、ADC_mask1_Volume、T2_mask1_10Percentile、T2_mask1_Energy、T2_mask1_Median、T2_mask1_Variance、ADC_mask2_TotalEnergy、ADC_mask2_Uniformity、

ADC_mask2_Variance、T2_mask2_Maximun、T2_mask2_Variance、ADC_mask3_10Percentile、ADC_mask3_Maximum、ADC_mask3_MeanAbsoluteDeviation、ADC_mask3_Range、ADC_mask3_Uniformity、T2_mask3_90Percentile、T2_mask3_Energy、T2_mask3_Minimum、T2_mask3_Range、T2_mask3_Variance

Features in figure b are shown from left to right:

ADC_1bp.3D.k_firstorder_Kurtosis、ADC_square_glszm_SizeZoneNonUniformity、ADC_wavelet.HHH_glcm_ClusterShade、ADC_wavelet.LHL_firstorder_Skewness、ADC_wavelet.LHL_glszm_LowGrayLevelZoneEmphasis、ADC_wavelet.LLH_firstorder_90Percentile、ADC_wavelet.LLH_firstorder_Maximum

Features in figure c are shown from left to right:

T2_1bp.3D.k_glcm_Correlation、T2_original_glrlm_LongRunHighGrayLevelEmphasis、T2_square_gldm_LargeDependenceHighGrayLevelEmphasis
